# Arterial stiffness by oscillometric device and telomere lenght in juvenile idiopathic artrhitis with no cardiovascular risk factors: a cross-sectional study

**DOI:** 10.1186/s12969-017-0165-1

**Published:** 2017-05-04

**Authors:** Maria Mercedes Picarelli, Luiz Cláudio Danzmann, Lucas Kich Grun, Nevton Teixeira Rosa Júnior, Patrícia Lavandovsky, Fátima Theresinha Costa Rodrigues Guma, Renato T. Stein, Florência Barbé-Tuana, Marcus Herbert Jones

**Affiliations:** 1 Rheumatology Department, Hospital São Lucas da Pontifícia Universidade Católica do Rio Grande do Sul (PUCRS), Ipiranga Avenue, 6690/220, Porto Alegre, 90610 000 Brazil; 2 Universidade Luterana do Brasil, Canoas, Brazil; 3 Universidade Federal do Rio Grande do Sul, Porto Alegre, Brazil

**Keywords:** Arterial stiffness, Pulse wave velocity, Telomere length, Artrhitis juvenile, Unifyng hypothesis

## Abstract

**Background:**

Advances in juvenile idiopathic arthritis (JIA) treatment is promoting free disease survival. Cardiovascular disease (CVD) may emerge as an important cause of morbidity and mortality. Pulse wave velocity (PWV), a surrogate marker of arterial stiffness, and telomere length (TL) are considered as potential predictors of CVD and its outcomes. The study aim was to assess PWV, TL in a JIA population and to test its correlation. In a cross sectional study, 24 JIA patients, 21 controls for TL and 20 controls for PWV were included. PWV was assessed by an oscillometric device. TL was assessed by qPCR. JIA activity was accessed by JADAS-27. Smoking, diabetes, obesity, renal impairment, hypertension, dyslipidemia and inflammatory diseases were excluded.

**Findings:**

Between cases and controls for TL, there was significant difference in age. No differences in gender, ethnics and bone mass index between JIA and control groups for PWV and TL. The JADAS-27 median was 8. TL was significantly reduced in JIA (0.85 ± 0.34 vs. 1. 67 ± 1.38, *P =* 0.025). When age adjusted by ANCOVA, the difference remained significant (*P* = 0,032). PWV was normal in all patients (5.1 ± 0.20 m/s vs. 4.98 ± 0.06 m/s, *P* = 0, 66). There was no correlation between TL, PWV or JADAS-27.

**Conclusion:**

Compared to controls, JIA with high disease activity and no CVD risk factors have shorter telomeres and normal PWV. As far as we know, this first time this correlation is being tested in rheumatic disease and in paediatrics.

## Background

Juvenile idiopathic arthritis (JIA) has chronic inflammatory activity as trademark of this condition [1]. As a result in the management and survival improvement in JIA, an increase in cardiovascular events is expected in this group of patients [1], as observed in other rheumatic diseases [2].

Patient’s surveillance in this context will have an emerging role. The evaluation of subclinical arterial disease is receiving greater attention, through different methods pointing to assess the presence of subclinical atherosclerosis and arteriosclerosis [3].

The increase in arterial stiffness is considered to be the main cause of systolic pressure elevation associated to aging and a risk predictor for cardiovascular events and mortality [4].

Pulse wave velocity (PWV) is seen currently as the simplest, non-invasive, robust and reproducible method to assess arterial stiffness, validated including in children [5].

Leukocyte telomere length (TL) is also been used to study biologic aging and cardiovascular senescence [6]. Telomeres are the tandem repeats of TTAGGG sequence at the ends of chromosomes [7]. Telomeres undergo shortening in their length every cycle cell [8] and are found shortened in many studies associated with cardiovascular disease [6] and autoimmune diseases [9].

Chronic inflammatory activity, oxidative stress and increased leukocyte renewal are present in autoimmune disease in general, as in JIA, and are appointed as the main environmental factors associated to telomere shortening [9] and impairment in endothelial function and vascular remodelling [10]. Therefore, the study aims were to 1) assess PWV and TL in a JIA group of patients; 2) test its correlation.

## Findings

In JIA patients, PWV is increased and TL is reduced, findings positively related to inflammatory activity.

JIA patients according to the 1997 ILAR criteria and controls for PWV were recruited at São Lucas Hospital, Pontifícia Universidade Católica do Rio Grande do Sul – (PUCRS), Porto Alegre – Brazil. We included patients beyond seven years old and with polyarticular rheumatoid factor positive and negative and oligoarticular persistent and extended subtypes. Obesity, smoking, systemic hypertension, dyslipidemia renal impairment, *diabetes mellitus* and other inflammatory diseases were excluded. For TL, controls were historical from previous studies from the same institution. The study was approved by the local ethics committee and an informed consent was obtained from all patients. Patients below 18 years old signed a free assent.

PWV was assessed by an oscillometric non invasive device through brachial artery occlusion (CardioSDyna – MAPA +). Patients followed the American Heart Association recommended instructions [3] for standard assessment of arterial stiffness. They were oriented to not use any vasoactive drugs 12 h before the evaluation and to avoid caffeine four hours before procedure.

For measures, patients rested in the supine position for ten minutes in a quiet room. Patients should not spoke during the measurement to prevent the influence of speaking on arterial tone. Evaluations were always in the morning to control for the diurnal variation of arterial stiffness. Measurements were always made by the same observer and two consecutive measurements were performed.

For TL, 10 ml of peripheral blood sample was collected in a matched way to the routine assessment, not representing additional procedure. qPCR was performed. Peripheral mononuclear cells were purified. Genomic DNA was extracted by phenol/chloroform/ isoamyl alcohol [11]. The relative telomere length was performed by calculating the ratio (T/S) of a quantitative PCR product from the same sample using specific primers for telomeres and a single copy gene as described by Cawthon [12].

Inflammatory activity was assessed by Juvenile Arthritis Disease Activity Score with 27 joints (JADAS-27) [13].

The Mann–Whitney test was used to compare healthy controls and JIA patients. Spearman test was used to test correlations between telomere length and JADAS, disease duration and pulse pressure. Pearson test was used to test correlations between telomere length, age and PWV. For controlling of confounders, Covariance analysis (ANCOVA) was used, with logarithmic transformation used on asymmetric data. *P* values less than 0.05 were considered significant.

Twenty-four JIA patients, twenty-one controls for TL and 20 controls for PWV were included. Between cases and controls for PWV, there was no significant difference in age, gender, ethnics and BMI. Between cases and controls for TL there was significant difference in age (15.5 ± 6.3 years vs. 11.4 ± 1.3 years, *P* = 0,005), no differences in gender, ethnics and BMI (Table 1). The JADAS median was 8 [0–20], indicating moderate to high disease activity. This result was more influenced by parents/patient global assessment of well-being by visual analog scale (Spearman test r = 0.872, *P* = 0, 001) than erythrocyte sedimentation rate (Spearman test r = 0.277, *P* = 0, 201). Oligoarticular subtype was 65.2% and there was no prematurity in the group (Table 1).

**Table 1:** Demographic and clinical data in patients with JIA and control for TL and JIA and controls for PWV

	JIA	Controls (TL)	*P*	Controls (PWV)	*P*
	n = 24	n = 21		n = 20	
Age (years)	15,5 ± 6,3	11,4 ± 1,3	0, 005^a^	13,8 ± 5,9	0,184^a^
Gender - n (%)					
Male	7 (26,1)	8 (40,9)	0,460^b^	6 (30)	0,458^b^
Female	17(73,9)	13 (59,1)		14 (70)	
Ethnics - n (%)					
Caucasian	19 (82,6)	17(81,8)	1, 000^c^	16 (80)	0,640^c^
Black	5 (17,4)	4 (18,2)		4 (20)	
Weight (Kg)	50,1 ± 17,5	42,5 ± 10,2	0, 085^a^	46,8 ± 13,7	0,528^a^
Height (m)	1,52 ± 0,17	1,47 ± 0,09	0, 162^a^	1,44 ± 1,09	0,473^a^
BMI (KG/m^2^)	20,7 ± 3,7	19,6 ± 2,7	0, 247^a^	18,2 ± 4,9	0,372^a^
Duration disease (years) [P25 - P75]	9 [5–19]				
Subtype – n (%)					
Oligoarticular	15 (65,2)				
Polyarticular	8 (34,8)				
Medications – n(%)					
Corticoids	8 (34,8)				
NSAIDS	0(0,0)				
Methotrexate	12 (52,2)				
Leflunomide	10 (43,5)				
Anti-TNF	7 (30,4)				
Uveitis – n (%)	1 (4,3)				

*NSAIDS* Nonsteroidal anti-inflammatory drugs, *Anti-TNF* Anti-tumour necrosis factor alpha agent

^a^ T-student test; ^b^ Pearson test; ^c^ Fisher test

No patient was receiving anti-inflammatory drugs. Disease modifying activity drugs were used in 95.7% of the group, about 30% were in corticosteroids use and the same proportion of patients was using biologic drugs, only Anti-TNF (Table 1).

PWV was normal in all patients, JIA and controls. The average PWV was 5.1 ± 0.20 m/s vs. 4.98 ± 0.06 m/s, *P* = 0,66). There was no correlation between pulse pressure and PWV (r = 0.172, *P* = 0. 434) and JADAS (r = 0,283, *P* = 0.191). There was a minimal variation between genders: 39.8 ± 8.68 mmhg among males and 38.8 ± 10.0 mmhg in females.

**Fig. 1:**
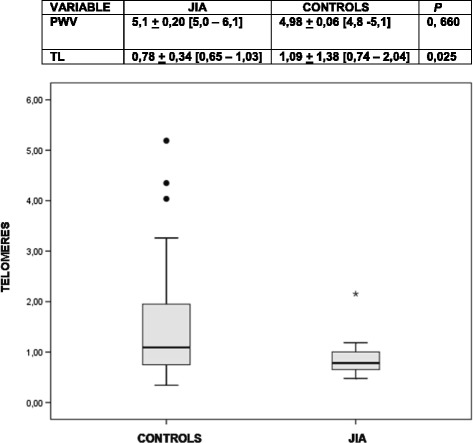
Telomere length in JIA and controls. Reference: relative telomere length between JIA patients and controls. There was significant difference between the groups (*P =* 0, 025) by Mann–Whitney test. When age adjusted by ANCOVA, the difference remains significant (*p* = 0, 032)

There was a significant difference in T/S ratio for telomere length between JIA patients and controls (0.85 ± 0.34 vs.1.67 ± 1.38, *P =* 0.025) (Fig. 1). When age adjusted by ANCOVA, the difference remains significant (p = 0, 032). There was no significant difference between JIA subtypes, gender and no association between T/S ratio and JADAS and disease duration. The coefficient of variation was 11%.

TL was shortened in JIA group of patients, oligoarticular and polyarticular subtypes, with long disease duration, high to moderate disease activity and no cardiovascular risk factors, compared to controls. There was no difference in PWV values among these groups and there was no correlation between TL and PWV in this group of JIA patients.

Our findings shown no evidence of correlation between TL and PWV, variables highly influenced by aging, in a JIA group of patients, composed by oligoarticular and polyarticular subtypes, long disease duration and disease activity considered as moderate to high.

As far as we know, this is the first study to test the correlations between those variables in rheumatic diseases and in paediatrics.

PWV and pulse pressure values were normal in JIA patients. In literature, we found five studies to evaluate PWV in JIA patients using different methods and with a high heterogeneity in subtypes selected [14–18] making comparisons difficult. We observed that studies that did not exclude cardiovascular risk factors had positive findings for PWV [14, 16–18]. Our findings are in agreement with the Vlahos *et al.* study, which excluded those factors, and found no difference in PWV values by tonometry. We believe these findings could be pointing to a greater role of atherosclerosis in JIA patients, but no arteriosclerosis. More studies are necessary to clarify this setting. We found no other studies to use oscillometry in JIA, a method highly applicable and with good correlations with other methods to assess PWV [5].

In relation to TL, Prelog *et al.* and Dvergstein *et al.* [17, 18] tested T cells and found significant differences in JIA compared to controls. Both authors believe there is evidence to indicate premature immunosenescence in JIA. Dvergstein *et al.* found a naive T cell compartment with shortened telomeres and reduced proliferative capacity in JIA. In the study of Prelog *et al.*, telomeric erosion was increased in CD4+, CD45RA+ with no correlations between age, sex, disease duration, use of methotrexate and corticosteroids, which are in agreement to our findings. In rheumatoid arthritis, a similar study also found no correlations between disease duration, disease activity index and therapy [9]. The authors support the idea that telomere shortening could be associated to an intrinsic defect in telomere maintenance or low activity of telomerase than to disease activity or immunosuppressive therapy. What is the meaning of telomere shortening in JIA remains a question to be answered for more studies, different than the cross-sectional design, which are majority in this area.

Are limitations of this study the small sample size. The significant difference in age (15.5 ± 6.3 vs.11.4 ± 1.3 years, *P = 0, 005*) might have been a potential bias, but the impact of this difference might not have been important since both groups had any participant below 7 years old, a crucial period in physiologic telomeric erosion process. The coefficient of variation was 11%, a limitation of qPCR method.

We believe this is an interesting *in vivo* model to study the inflammatory activity influence on those variables, excluding the impact of aging and cardiovascular risk factors, as potential confounders. The exclusion of patients below seven years old had also removed from the study group, patients that could have their PWV values in physiologic elevation process and their TL also in physiologic shortening, both phenomena associated to growing.

## Abbreviations

ANCOVA: Covariance analysis
CVD: Cardiovascular disease
JADAS-27: Juvenile arthritis activity score, counting 27 joints
JIA: Juvenile idiopathic arthritis
PWV: Pulse wave velocity
qPCR: Quantitative proteinase chain reaction
TL: Telomere length
